# Challenges of Reproductive Health Management in the Camps of Internally Displaced Persons: A Systematic Review

**DOI:** 10.4314/ejhs.v31i1.20

**Published:** 2021-01

**Authors:** Farin Fatemi, Shandiz Moslehi

**Affiliations:** 1 PhD, Research center for health sciences and technologies, Semnan University of Medical Sciences, Semnan, Iran; 2 PhD, Health Management and Economics Research Center, Iran University of Medical Sciences, Tehran, Iran

**Keywords:** Internally Displaced Persons, Reproductive Health, Humanitarian Assistance, Humanitarian Settings, Disasters

## Abstract

**Background:**

Internally Displaced Persons (IDPs) in the camps face many reproductive health challenges. They should meet their needs timely to save their lives. This paper outlines a systematic review to discuss the challenges of reproductive health management in the camps of internally displaced persons.

**Methods:**

For this research, electronic databases including PubMed, Science Direct, Scopus, Pro Quest, Google Scholar and Cochrane Library till January 1, 2020 were searched. A threestage screening process was used for the selection of literature due to PRISMA checklist. Finally, a thematic synthesis approach was applied to analyze the data.

**Results:**

In total, 133 articles were identified; 11 articles met the inclusion criteria for entering the process of final analysis. The findings were demonstrated in six main categories of availability and accessibility of reproductive health services, sexual and gender-based issues, human rights, social and cultural issues, coordination and collaboration, and mental health issues. The remarkable result of this study highlighted that the main challenges are lack of access to health services, violence against women and lack of household education.

**Conclusion:**

Results of this systematic review present valuable advice for policy makers and managers to prepare and respond effectively and timely to reproductive health challenges of internally displaces persons. Disaster preparedness plans and contingency plans for maintaining and developing reproductive health in IDPs camps are recommended.

## Introduction

Every year, millions of people around the world miss their homes as a result of conflicts and disasters and become internally displaced within their own countries (1). A total of 41.3 million people were estimated to be living in internal displacement at the end of 2018 (2).

Internally Displaced Persons (IDPs) have the same health needs as non-displaced people. Women accounted for around half of the IDPs in 2010 (3). They are usually both the primary caretakers of children and siblings and the providers of family income; their multiple responsibilities make it hard for them to access education or health services (4). Additionally, women and girls often face increased risk of violence and may be unable to access assistance or make their health needs met (5,6). Todays, women and children constitute around 80% of IDPs, and international humanitarian agencies have recognized the special needs (7). Meeting the sexual and reproductive needs of IDPs, particularly the female ones is part of a process of restoring their lost rights of citizenship (4).

Reproductive health is a state of complete physical, mental and social well-being and not merely the absence of disease and infirmity, in all matters related to the reproductive system and to its functions and processes (3). Many studies have indicated that the reproductive health needs of adolescents have not been met appropriately due to limited resources and existing challenges in IDPs camps (8). Adolescents in camps are exposed to sexual activities, both consensual and forced, without the use of contraceptives that result in unintended pregnancies, STIs, unsafe and spontaneous abortion and other reproductive health risks (9). These distressing events happen because the reproductive health care is associated with a wide range of challenges and problems. Lack of access to IDPs camps, politically or geographically, insufficient funds and staff or security issues are the instances of difficulties that humanitarian agencies face during serving to IDPs (10). Therefore, using a multi-sectoral approach, involving protection, security, community and health sectors is necessary to effectively respond to reproductive health needs of IDPs in disasters and emergencies (3,6). Having this approach is necessary to find the probable challenges as many as possible. Then, the multi-sectoral approach should be organized with considering the extracted challenges for delivery of reproductive health care in IDPs camps.

The purpose of this systematic review study is to determine the reproductive health challenges of IDPs in disasters and emergencies.

## Methods

**Search strategy and selection criteria**: Six electronic databases were searched to identify studies from published and grey literature on productive health problems and challenges of IDPs. The populations of interest were persons who had been internally displaced. The search was done on January 1, 2020 and not limited to a specific time frame. The bibliographic databases searched were PubMed, Scopus, Science direct, ProQuest, Cochrane Library for English literature and Google Scholar for Persian ones. The search terms adopted include: (“Internally displaced person*” OR “Conflict-affected persons”) AND (“Reproductive health”) AND (Challenge* OR Problem*) AND (Disasters). Reference listings of identified articles were also independently hand-searched for more specific articles. The numbers of articles/abstracts generated from the various databases are indicated in Table 1.

**Table 1:** Databases searched and number of founded articles

Name of Database	Number of articles
PubMed	12
Scopus	20
Science Direct	46
ProQuest	55
Cochrane Library	0
Google Scholar	0

***Total***	*133*

**Figure 1:**
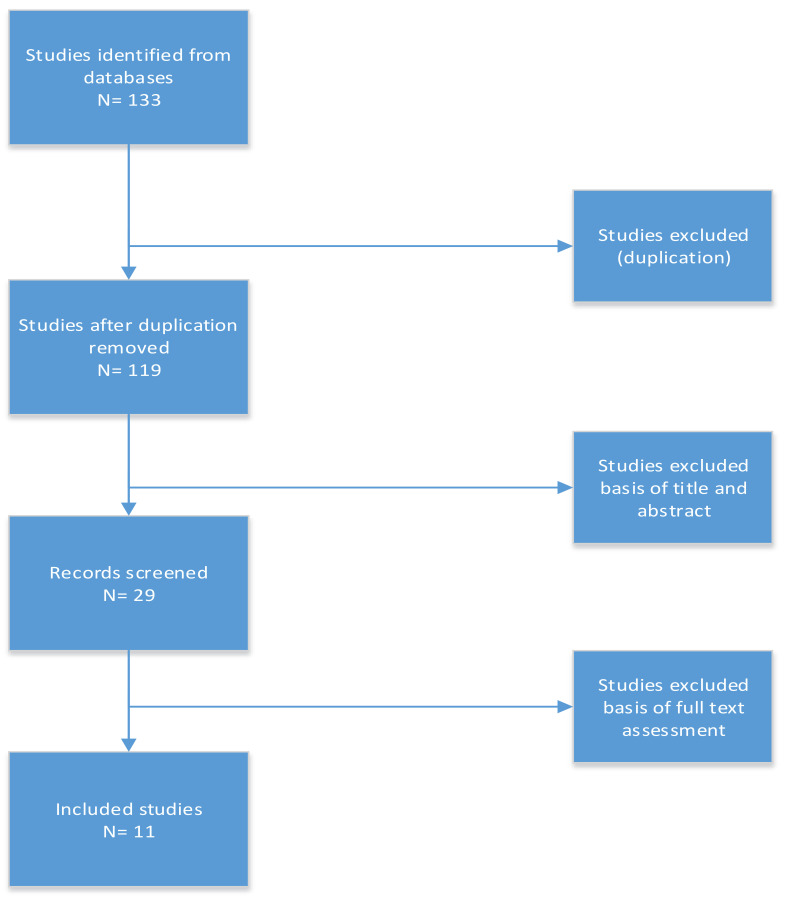
Flow diagram of screening process for review

**Study screening and selection**: A three-stage screening process was undertaken for the selection of literature for the study. Initially, the authors conducted independent searches based on the search strategy. Secondly, title and abstract of founded articles were screened independently by the authors to assess their eligibility for inclusion in the review. This stage was conducted using the inclusion and exclusion criteria. Finally, the available full texts of the selected articles were reviewed to confirm that the studies met the research question of this review. The process for selecting and reviewing the articles has been indicated in Figure 1.

**Inclusion and exclusion criteria**: All the studies with different study designs and methodologies that reported the reproductive health challenges of IDPs were included. Studies with no data on the scope of the research question of this review were excluded alongside books, guidelines, peer-review and online articles and reports. Also, the articles that whose full texts were not available or were in other languages other than English and Persian were excluded. Furthermore, time, age and sex were not factors considered for exclusion in this study.

**Data extraction**: The full texts of eligible studies for this review were independently screened and summarized according to designed forms for descriptive and thematic analysis. Information about the author, country in which the study was carried out, study year, study design, objectives and key study results were extracted from the descriptive analysis. Another form was applied for extracting the challenges of reproductive health in IDPs and categorizing them. Finally, the manuscript was evaluated by PRISMA checklist.

## Results

The screening process yielded a total of 133 citations/abstracts (Stage 1). The duplicated studies were removed, and of 119 studies after reviewing the titles and the abstracts, a total of 90 of these were rejected because they were not relevant to reproductive health challenges of IDPs, or the study populations were done in either refugees or unrelated populations (Stage 2). A total of 29 articles were left for the full-text review (Stage 3). Subsequently, 18 studies were discarded because they did not meet the inclusion criteria (Figure 1).

**Descriptive results**: By reviewing the eleven included articles, it was declared that the largest numbers of papers were from Africa (36%), followed by Asia (18%) and United States (18%). Other studies were from Europe and South America. The descriptive analyses of the most important included articles in this study are summarized in Table 2.

**Table 2:** Characteristics and findings of studies on reproductive health challenges in IDPs

Author (year)	Country	Study design	Objectives	Key findings
**Sahoo &** **Pradhan,** **(2018)**	India	Literature review	Looking into the IDPs reproductive healthcare situation in India	IDPs were susceptible to a number of health problems due to the exposure to physical and environmental threats, violence and trauma.
**Nidzvetska** **et al, (2017)**	Ukraine	Cross-sectional	Exploring health problems of mothers and young children displaced by the conflict in Ukraine	Increasing social allowances and their timely delivery to IDP mothers might be the most efficient policy measure to improve health
**Westhoff et** **al, (2008)**	United States	Cross-sectional	Examining reproductive health care indicators among refugees and IDPs in southern Belize	Better access to medical services and education about reproductive health issues are needed in following disasters.
**Parker et al,** **(2014)**	Uganda	Cross-sectional	Investigating the menstrual management challenges in Uganda	There was a lack of education about reproductive health and practices are strongly influenced by cultural taboos.
**Orach et al,** **(2009)**	Uganda	Cross-sectional	Exploring IDPs access to health services and gender-based violence	Main constraints to population access to health care included lack of money and lack of information
**Casey (2016)**	Democratic Republic of the Congo	Cross-sectional	Addressing the gaps for the implementation of contraceptive services in humanitarian settings	These sexual and reproductive health programs should be implemented by multi-sectrol in full collaboration with the Ministry of Health (MOH) and health workers.
**Balinska et** **al, (2019)**	Lebanon and Iraq	Cross-sectional	Investigating the reproductive health profile of all women in Lebanon and Iraq	- Pregnancy was unplanned for 57% and 66.7% of women who had delivered in the previous year. Pregnancy in multivariable analysis.
**Larrance et** **al, (2007)**	United States	Cross-sectional	Assessing health needs of IDPs	Intimate partner violence and suicide completion rates rates post displacement were higher than US baseline rates.

**Analysis**: Classification of reproductive health management challenges of IDPs in disasters are shown in Table 3. A thematic synthesis approach was used to gather information, and inductive analysis was performed by the two authors. For designing this table, the authors extracted the findings and coded the findings of each study, then grouped the codes due to their similarity and finally analyzed the grouped findings to classify them. The accuracy and completeness of the extracted data were checked by the two authors.

**Table 3:** Classification of reproductive health management challenges of IDPs

Category	Subcategory	Reference(s)
***Availability and*** ***accessibility of*** ***reproductive health*** ***services***	Lack of availability of reproductive health services	(5, 13, 21)
	Neglected family planning	(12, 17, 19)
	Unplanned pregnancy	(11, 14, 17)
	Lack of addressing reproductive health needs	(11, 12)
	Lack of access to certain services	(11–16)
	Increase preventable maternal and infant death	(17)
	Lack of referral services	(16)

***Sexual and gender-based*** ***issues***	Spread of HIV	(12, 17)
	Violence against women/ rape/ sexual abuse	(12, 15–18)

***Human rights***	Dignity	(16, 20, 21)
	Equity	(16)

***Social and Cultural issues***	Attitude, cultural taboos, religious and community beliefs	(17, 19, 20)
	Lack of household education	(11, 12, 14, 15, 19, 20)
	Lack of community awareness	(16)

***Coordination and*** ***Collaboration***	Lack of finance	(16)
	Lack of Information	(16)
	Lack of skilled staff	(16)
	Lack of adequate standards	(21)
	Lack of accountable authorities	(13)

***Mental health***	Lack of psychological support	(15)
	Depressive disorder	(18)
	Suicide-attempt	(18)

The database search yielded 101 papers (Figure 1). Of these, 11 papers met the inclusion criteria and were included in the synthesis. Papers were screened out following full-text assessment owing to: lack of empirical data, out of study scope modality, and/or a non-humanitarian setting context. None of the grey literature sources met our inclusion criteria.

The 11 included studies reported on reproductive health management in IPDs focusing on lack of access to certain services (11–16), violence against women (12,15–18), lack of education (11,12,14,15,19,20), attitude, cultural taboos, religious and community beliefs (17,19,20), human rights and dignity (16,20,21), neglected family planning (12,17,19) and unplanned pregnancy (11, 14, 17).

The main challenges were described and categorized in the field of reproductive health management in the humanitarian settings for IDPs at this stage. Availability and accessibility of reproductive health services, sexual and gender-based issues, human rights, social and cultural issues, coordination and collaboration, and mental health issues were the main categories (Table 1) (22,23). Finally, 22 challenges were identified from the literature. Seven subcategories (31.8%) for availability and accessibility of reproductive health services, two subcategories (9%) for sexual and gender-based issues, two subcategories (9%) for human rights, three subcategories (13.7%) for social and cultural issues, five subcategories (22.8%) for coordination and collaboration, and three subcategories (13.7%) for mental health issues. The majority of the challenges were dedicated to the category of availability and accessibility of reproductive health services.

## Discussion

This review identified a limited evidence base on the main IDPs challenges of reproductive health management in the humanitarian settings with available studies covering divergent settings. Available studies revealed that there are many fundamental challenges to IDPs reproductive health management (19,21).

**Availability and accessibility of reproductive health services**: The first important challenge in this category was lack of access to certain services (11–16). Lack of access to humanitarian assistance was generally discussed in different studies (24–27), but some of them were focused on access to reproductive health services (28). Neglected family planning (12,17,19), unplanned pregnancy (11,14,17), preventable maternal and infant death (17), spread of HIV (12, 17) and violence against women (12, 15–18) may be the results of lack of access to certain services.

A woman has many reproductive health needs, through the different stages of reproductive life such as access to a full range of family planning services, safe abortion care, and prenatal, delivery and postnatal care (29). Reproductive health needs should be recognized and addressed in certain services (11,12). The needs may be different in each humanitarian setting. Thus, it should be studied and defined. This fits previous findings (30–34). Addressing preparedness activities, re-establishing or strengthening reproductive health, preventing gender-based violence, setting up tents to deliver reproductive services in remote areas are some examples of different reproductive needs (35).

There are some health concerns of women and infants in disasters (36). Unplanned pregnancy, preventable maternal and infant death are main concerns in this issue (37). Limited access to reproductive services may cause unplanned pregnancy. Inequity in distribution of clean water and safe food, interruption of health services, crowded shelters and camps and limited access to reproductive health care may threaten the health of pregnant women (36); so it may lay to maternal and infant death.

**Sexual and gender-based issues**: In disasters and emergency situations, overall levels of violence increase (38). Women and children are vulnerable to this violence. Now, there are some organizations, international safe communities and NGOs which support the women and children. Also, the United Nations Sustainable Development Goals (UNSDG) increase the interest of communities to take actions in preventing violence against women (39). Community members, policy makers, the police, case managers, trauma psychologists, family violence workers (40) and integration of disaster management and women's services programs play an important role in this regard (41).

**Human rights**: Some studies mentioned that women in IDP camps suffer diminished human rights and dignity (20). This is because of women lack knowledge about their rights, inadequate national laws and absence of female officers in the disaster area (37). National policy makers should protect vulnerable women and pay attention to the reproductive health needs, rights and dignity of IDPs (13). Humanitarian law, human right, and international criminal law are some examples of actions through increasing the women rights and dignity (42). Also, Sustainable Development Goals 3, 5 and 16 mentioned the rights of women (37).

**Social and cultural issues**: Lack of household education is one of the main reproductive health management challenges of IDPs in disasters. Most of the studies had focused on this issue (11,12,14,15,19,20). Some studies have mentioned that household education level is positively related to disaster preparedness and disaster related education can increase the personal preparedness, which is crucial in mitigating the risks (43). Disaster related education can be developed in primary school, secondary school, and within the community to further build resilience to disasters (44). To assess the current level of community resilience, factors such as education play an important role (45). Therefore community education is also another subject of study in this regard. Religious places such as churches can play an important role to promote the education of the community about disasters (46).

Women in IDP shelters suffer from reproductive health challenges and their activities are mainly influenced by cultural taboos and community beliefs (20). The health care stakeholders should ensure women's access to healthcare services. Thus, they may focus on improving the community education. Religious places and groups have a great potential in improving the community education. If the policy makers increase the contribution of such places and groups in preparedness and mitigation activities, they can use their potential of these groups in response and recovery phases of disaster management (47).

**Coordination and collaboration**: One of the challenges was lack of adequate standards for managing the reproductive health. One of the active roles of the government and humanitarian assistance organizations is to provide adequate standards of living and social security for IDPs. This is in complete agreement with previous studies (21). Minimum Initial Service Package (MISP) is one of the standards in this regard which can be used in humanitarian settings to meet the needs of IDPs (48–50). Developing standards can increase the preparedness of governments and humanitarian organizations. Thus, in disasters and emergencies, there is a great need of such standards to be stablished and used. This is because women and girls are at the highest risk for maternal mortality due to the unhealthy timing and spacing of pregnancies and giving birth (51). Also, monitoring and evaluation of implementation of these standards should be considered. Importance of monitoring the standards is found in previous studies (50).

**Mental health issues**: Mental disorders after disasters reported in previous studies. Depression especially in women are recognized as a common mental disorder. Post disaster suicide-attempt rates and depressive disorders are higher in internally displaced population (18). Violence against women, rape, sexual abuse and unplanned pregnancy can make the situation worse. Using shelter surveillance system, improving security (18), and access to mental healthcare can be used as preventive actions in this regard.

In general, IDPs in camps face with many reproductive health challenges. In this systematic review, we provided a comprehensive discussion and summarized all aspects of these challenges. Developing disaster preparedness and contingency plans, estimating the immediate reproductive health needs using the MISP, increasing the knowledge of women about their rights, and addressing the mental and psychosocial needs are important approaches to decrease these challenges. Also, planning for gender-based violence prevention, developing referral programs, using female officers in the IDP camps, creating standards of living and social security for IDPs and increasing the access to a full range of family planning services are some recommendations to decrease the reproductive health management challenges of IDPs in disasters.
